# Interfacial Defect Suppression and Enhanced Optical Properties in InP Quantum Dots via Two-Step ZnSe Shelling Strategy

**DOI:** 10.3390/ma18174172

**Published:** 2025-09-05

**Authors:** Jaehyeong Yoo, Sung-Yoon Joe, Jae-Hyeon Ko

**Affiliations:** School of Nano Convergence Technology, Nano Convergence Technology Center, Hallym University, Chuncheon 24252, Gangwondo, Republic of Korea; dbwogud0721@naver.com (J.Y.); starlitjay@gmail.com (S.-Y.J.)

**Keywords:** InP quantum dots, 2-step ZnSe shelling, 1-step ZnSe shelling, oxygen trap, Interface defect

## Abstract

This study investigates the interfacial structural origin of enhanced optical performance in InP-based quantum dots (QDs) employing a 2-step ZnSe shelling strategy. By comparing InP/ZnSe/ZnS QDs synthesized via 1-step and 2-step shelling methods using identical InP cores, we demonstrate that the 2-step approach results in improved core–shell lattice matching, more favorable carrier dynamics, and enhanced thermal stability. These enhancements are attributed to the formation of an initial thin ZnSe interfacial layer, which facilitates uniform shell growth and suppresses interfacial defect formation. High-resolution transmission electron microscopy and elemental mapping via energy-dispersive X-ray spectroscopy analyses confirm the improved crystallinity and reduced oxygen-related trap states in the 2-step samples. The findings highlight the critical role of interfacial control in determining QD performance and establish the 2-step ZnSe shelling strategy as an effective route to achieving structurally and optically robust QD emitters for advanced optoelectronic applications.

## 1. Introduction

III–V-based quantum dots (QDs) have attracted considerable attention for applications in displays and optoelectronic devices due to their broad tunability in emission wavelength and excellent photoluminescent properties. Among them, environmentally friendly indium phosphide (InP) QDs have emerged as promising emitters for next-generation display technologies, which demand high efficiency and narrow-band emission for enhanced brightness and color purity [1,2,3]. The photoluminescence (PL) performance of InP QDs has been steadily improved through various ligand treatments and surface treatment techniques [4,5,6]. However, InP cores remain vulnerable to intrinsic and extrinsic defects. To overcome these limitations, the growth of inorganic shells—particularly ZnS-based shells—has been widely employed [7,8,9]. In particular, double-shell structures have been reported to offer superior optical stability and higher emission efficiency compared to their single-shell counterparts, as they can effectively suppress interfacial defects and relieve lattice mismatch-induced interfacial strain. Such double-shell configurations typically employ ZnSe (5.668 Å) as an intermediate shell to improve crystallinity, bridging the large lattice mismatch between the InP core (5.868 Å) and the outer ZnS shell (5.411 Å) and simultaneously enhancing quantum confinement via a stepped energy band structure [10,11]. Nevertheless, defects introduced during the shelling process remain a major factor limiting the optical performance of InP-based QDs. These defects form nonradiative recombination pathways, thereby reducing the PL quantum yield (PLQY) and impairing the optical stability of the material [12,13]. In particular, for unstable materials such as InP, the degree of interfacial defects is highly sensitive to the shell growth method, which in turn significantly influences the final optical properties.

These defect-related issues are not limited to surface states but are also associated with structural instabilities at the core–shell interface. During shell growth, chemical and structural mismatches between the InP core and the shell can give rise to structural defects, which in turn can accelerate surface oxidation. In particular, InP is prone to oxidation upon exposure to ambient conditions, leading to the formation of nonradiative recombination pathways. Such nonradiative recombination ultimately results in a decrease in PLQY [14]. Indeed, Moriyama et al. investigated the impact of interfacial oxygen concentration on the PLQY and lifetime of InP QDs and reported that the interfacial quality is critically linked to the overall optical performance [15]. Therefore, a shell growth strategy capable of simultaneously mitigating interfacial defects is required. To address these challenges, recent studies have introduced stepwise injection shelling strategies, in which shell precursors are gradually and sequentially introduced in two stages. This approach has been employed to promote the formation of uniform and highly crystalline shells, while effectively suppressing interfacial defects and oxidation of the InP core [16,17].

In this study, we investigated InP/ZnSe/ZnS QDs synthesized using a common InP core, with two different ZnSe shell growth methods: a stepwise injection shelling strategy (InP/2-step ZnSe/ZnS) and a single-injection shelling strategy (InP/1-step ZnSe/ZnS). The QDs prepared via the 2-step ZnSe shelling process exhibited better structural properties compared to those synthesized by the 1-step method, particularly in terms of improved core–shell lattice matching and the associated suppression of interfacial defects. These improvements are attributed to the suppression of nonradiative recombination pathways, such as void formation and oxygen-related trap states, which are typically induced by interfacial defects at the core–shell boundary. However, only a few studies have rigorously examined the effects of the 2-step ZnSe shelling process at the interfacial level through detailed structural and chemical analyses and correlated them directly with the enhanced optical properties. In this study, we quantitatively analyzed the relationship between interfacial quality and optical performance of QDs synthesized via the two different shelling methods, using measurements of PLQY, temperature-dependent PL, time-resolved photoluminescence (TRPL), high-resolution transmission electron microscopy (TEM), and elemental mapping via energy-dispersive X-ray spectroscopy (EDS). The results demonstrate that the 2-step ZnSe shelling strategy significantly improves the structural integrity and chemical stability of the core–shell interface, thereby serving as an effective approach for enhancing the optical performance of InP-based QDs.

## 2. Materials and Methods

### 2.1. Materials

InP core QDs (emission peak at 570 nm) were purchased from Uniam (Republic of Korea). Trioctylamine (TOA), trioctylphosphine (TOP), sulfur (S), selenium (Se), zinc acetate, oleic acid (90%), and hydrofluoric acid (HF) were purchased from Sigma-Aldrich (St. Louis, MO,USA) and used as received without further purification. All syntheses were carried out using a Schlenk line system under vacuum and a nitrogen atmosphere to exclude oxygen and moisture.

### 2.2. Precursors

TOP-Se and TOP-S precursors were prepared by dissolving Se and S in TOP at concentrations of 64 mg/mL and 158 mg/mL, respectively, followed by stirring at 60 °C for 24 h. The Zn precursor (Zn(OA)_2_) was prepared by dissolving zinc acetate and oleic acid in TOA at 110 °C until fully dissolved, forming a homogeneous Zn(OA)_2_ precursor solution.

### 2.3. 2-Step ZnSe Shelling

Initially, Zn(OA)_2_ and a small amount of Se precursor (0.4 mmol) were sequentially injected into the reactor containing the TOA solvent and allowed to react with the InP core. Subsequently, hydrofluoric acid (HF, 0.1 mL) was added prior to the main shelling process. HF has been reported to passivate the InP core surface by removing surface oxides and suppressing trap states [18,19,20]. The mixture was then heated to 220 °C and maintained under a nitrogen atmosphere for approximately 30 min. During this step, the small amount of Se precursor partially reacted at 220 °C, forming a thin Se interfacial layer. Afterward, an additional 1.0 mmol of Se precursor was injected to initiate ZnSe shell growth.

### 2.4. 1-Step ZnSe Shelling

The 1-step ZnSe shelling process omitted the initial thin-shell formation step used in the 2-step method. Instead, a total of 1.4 mmol of Se precursor was injected at once under the same reaction conditions, and the temperature was rapidly increased directly to 320 °C without the intermediate 220 °C step. The ZnSe shell was then grown in a single step. All other reaction conditions and procedures were identical to those used in the 2-step method.

### 2.5. ZnS Shelling

For the outer ZnS shell, identical shelling conditions were applied to both samples. An initial 1.6 mmol of S precursor was injected at 320 °C and allowed to react for 40 min to form a thin ZnS layer. The reaction temperature was then lowered to 280 °C, followed by an additional injection of 3.39 mmol of S precursor. The reaction was continued for 1 h to complete the ZnS shell growth. The resulting QDs were cooled to room temperature and purified by centrifugation at 4000 rpm for 10 min using a mixed solvent of acetone and toluene. The precipitated QDs were then redispersed in toluene at a concentration of 100 mg/mL, completing the synthesis.

### 2.6. Characterization 

UV–Vis absorption spectra were obtained using a spectrophotometer (X-ma 3000PC, Human Corporation, Seoul, Republic of Korea), and PL spectra were recorded with a fluorescence spectrometer (FS-2, Scinco, Seoul, Republic of Korea). Both optical measurements were carried out on QDs dispersed in toluene. PLQY was determined using a spectrophotometer ((QE-2100, Otsuka Electronics, Osaka, Japan) at Uniam with an excitation wavelength of ~450 nm and a sample absorbance range of 0.2–0.4. For each sample, six measurements were performed, and the average values were reported together with standard deviations as error bars to ensure reliability. TRPL measurements were conducted using a fluorescence spectrometer (FluoTime 300, PicoQuant GmbH, Berlin, Germany) under the same experimental conditions described in ref. [21]. High-resolution TEM (HR-TEM) imaging and energy-dispersive X-ray spectroscopy (EDS) elemental mapping were carried out on thin-film samples using an electron microscope (JEM-ARM200F, JEOL Ltd., Tokyo, Japan) operated at 200 kV and equipped with an Oxford Instruments X-Max EDS detector.

## 3. Results and Discussion

### 3.1. Optical Characteristics

**Figure 1:**
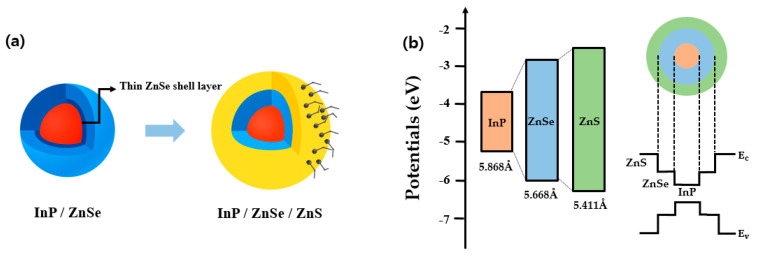
(**a**) Schematic representation of the core/shell structure of InP/2-ZnSe/ZnS quantum dots synthesized through a 2-step ZnSe shell growth method. (**b**) Energy band diagram of the InP/2-ZnSe/ZnS quantum dots. *E_c_* and *E_v_* denote the conduction band minimum and the valence band maximum of ZnS, respectively.

The introduction of a ZnSe/ZnS double-shell structure in InP-based QDs offers significant advantages in terms of charge confinement and interfacial stability. Such double-shell structures composed of ZnSe and ZnS, as illustrated in Figure 1, create a stepped energy barrier that effectively confines photoexcited electrons and holes in the InP core, thereby enhancing radiative recombination efficiency. This quantum confinement effect leads to an enhancement in PLQY [22,23].

In addition, incorporating ZnSe (5.668 Å) as an intermediate shell—whose lattice constant more closely matches that of InP (5.868 Å) than that of ZnS (5.411 Å)—can effectively alleviate strain and interfacial defects arising from the lattice mismatch between InP and ZnS. This contributes to improved interfacial stability and a reduction in nonradiative recombination pathways [24,25].

Building upon these structural advantages, the present study quantitatively investigates the effects of a stepwise ZnSe shell growth strategy on structural integrity and performance enhancement. The stepwise injection shelling method is employed as a strategy to mitigate interfacial stress during shell growth, thereby suppressing defect formation and minimizing nonradiative pathways such as interfacial dislocations and void formation. We quantitatively analyzed how this approach influences the structural stability and optical performance of InP-based QDs.

#### Photoluminescence Efficiency

**Figure 2:**
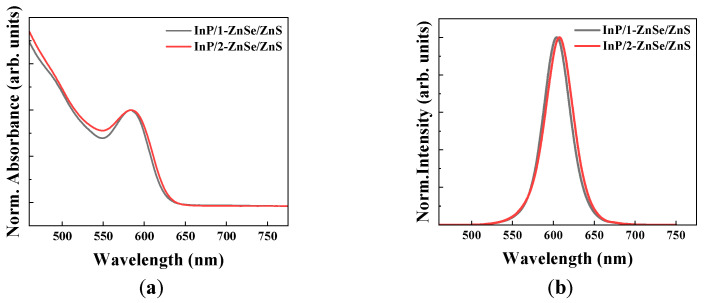
(**a**) Normalized absorbance of InP/ZnSe/ZnS quantum dots synthesized via 1-step ZnSe shelling and 2-step ZnSe shelling methods. (**b**) Normalized photoluminescence of InP/ZnSe/ZnS quantum dots synthesized via 1-step ZnSe and 2-step ZnSe shelling methods.

Figure 2a,b presents a comparison of the normalized absorbance and PL spectra, respectively, for two types of QDs prepared under different shelling conditions. The absorbance spectra were measured using a UV–Vis spectrophotometer (X-ma 3000PC, Human Corporation, Seouol, Republic of Korea), and the PL spectra were recorded with a fluorescence spectrometer (FS-2, Scinco, Seoul, Republic of Korea). Both measurements were performed for QDs dispersed in toluene solution. In addition, for PLQY measurements, six repeated measurements were performed for each sample to ensure reliability, and the average values with standard deviations were reported. Table 1 summarizes the peak wavelength, full width at half maximum (FWHM), and PLQY of the two types of QDs. The InP/1-step ZnSe/ZnS QDs and InP/2-step ZnSe/ZnS QDs exhibited emission peaks at 603.9 nm and 607.6 nm, FWHMs of 38 nm and 39 nm, and PLQYs of 94.0% and 95.7%, respectively, demonstrating excellent optical performance of the two samples. The double-shell structure appears to enhance the crystallinity of the shell layer and contributes to the narrow FWHM and slightly high PLQY by promoting uniform particle size distribution [24].

In particular, the 2-step ZnSe-shelled QDs prepared via the stepwise injection method exhibited a high PLQY of 95.7% and red shifts in both absorption and emission spectra, indicating that radiative recombination was effectively facilitated through the suppression of interfacial defects and oxidative trap states [26]. These results demonstrate that the 2-step ZnSe shelling strategy may play a critical role not only in improving structural stability but also in enhancing optical performance.

### 3.2. Time-Resolved Photoluminescence Characteristics

**Figure 3:**
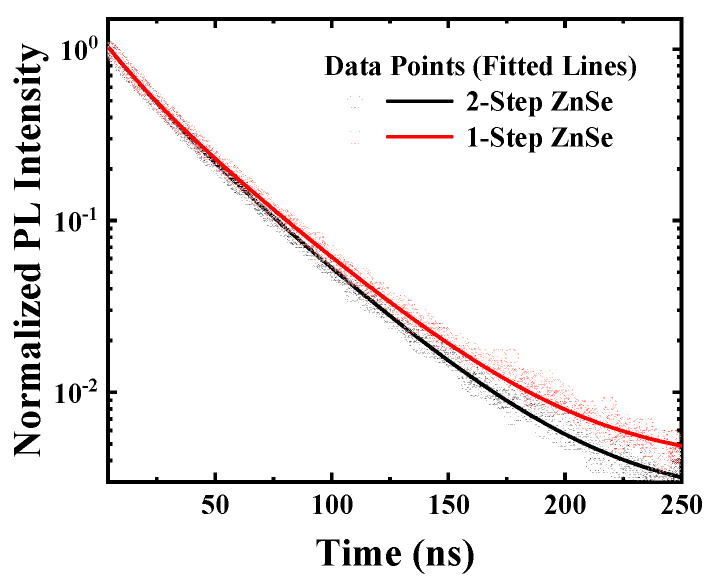
TRPL decay curves of 1-step and 2-step ZnSe-shelled QDs fitted with a double-exponential function expressed by Equation (1).

However, the previously discussed enhancement in emission efficiency cannot be solely attributed to the mitigation of interfacial defects. Therefore, to gain insight into the dynamic behavior of charge carriers and to analyze recombination characteristics at the core–shell interface, TRPL measurements were performed. Figure 3 presents the TRPL decay curves for the 2-step ZnSe and 1-step ZnSe samples, along with the results of bi-exponential fitting. The decay profiles were fitted using the following Equation (1):

(1) $$y=a_{1}exp\left(\frac{-t}{\tau_{1}}\right)+a_{2}\exp\left(\frac{-t}{\tau_{2}}\right),\;\tau_{avg}=\frac{{a_{1}\tau }_{1}^{2}+{a_{2}\tau }_{2}^{2}}{a_{1}\tau_{1}+a_{2}\tau_{2}}$$

In this equation, *y* denotes the PL intensity at time *t*, which is the elapsed time after photoexcitation. *a*_1_ and *a*_2_ represent the relative contributions of the fast and slow decay components, respectively [27,28]. τ_1_ is the lifetime of the fast decay component, typically associated with nonradiative, trap-assisted recombination processes. τ_2_ corresponds to the lifetime of the slow decay component, generally attributed to radiative exciton recombination. The average carrier lifetime, $\tau_{avg}$, is calculated as the amplitude- and time-weighted mean of the two decay components.

TRPL decay curves serve as an indicator for analyzing charge carrier recombination pathways within quantum dots and are commonly fitted using a bi-exponential function. In this fitting, the fast decay component ($\tau_{1}$, *a*_1_) is typically attributed to trap-assisted nonradiative recombination, reflecting rapid carrier quenching due to trapping at interfacial or defect states. In contrast, the slow decay component ($\tau_{2}$, *a*_2_) corresponds to radiative recombination processes [29]. The fitting results are summarized in Table 2.

According to the results presented in Table 2, the fast decay component of the 1-step ZnSe-shelled QDs exhibited τ_1_ = 14.34 ns and *a*_1_ = 0.28, which were lower than those of the 2-step ZnSe-shelled sample. This indicates that charge carriers were more readily trapped at interfacial defects, leading to rapid nonradiative recombination. Such behavior is attributed to the formation of interfacial defects during shell growth in the 1-step structure, likely due to the lack of control over the initial interface formation.

In contrast, the 2-step ZnSe-shelled QDs exhibited τ_1_ = 16.94 ns and *a*_1_; = 0.31, indicating a relatively slower fast decay component. This suggests that trap-assisted nonradiative recombination was mitigated to some extent. The improved performance is interpreted as the result of the initial formation of a thin ZnSe layer in the 2-step shelling strategy, which promotes uniform shell growth and effectively suppresses surface defects. Consequently, fewer excitons are lost through nonradiative pathways.

In addition, the slow decay component of the 1-step ZnSe-shelled sample was τ_2_ = 37.19 ns and *a*_2_ = 0.72, whereas that of the 2-step ZnSe-shelled sample was τ_2_ = 36.28 ns and *a*_2_ = 0.69, slightly lower than that of the 1-step case. This difference can be interpreted as a consequence of suppressed interfacial defects, which allow charge carriers to be more effectively confined rather than being lost to trap states, thereby promoting the radiative recombination previously discussed in the fast decay component.

As a result, although the average carrier lifetime ($\tau_{avg}$) of the 1-step ZnSe-shelled sample (34.20 ns) was slightly higher than that of the 2-step ZnSe-shelled sample (32.93 ns), the relative contribution of the fast decay component was more pronounced in the 1-step case. This implies that in the 2-step ZnSe-shelled QDs, charge carriers are less likely to undergo defect-mediated nonradiative recombination and are instead more efficiently preserved for radiative recombination. This interpretation is consistent with the higher PLQY (95.7%) observed in the 2-step ZnSe-shelled sample.

### 3.3. Temperature-Dependent Photoluminescence

These structural differences may also influence the thermal stability of the PL signal under elevated temperatures. Interfacial defects can be thermally activated, causing electron–hole carriers to escape via nonradiative pathways, thereby resulting in a decrease in PL intensity. Temperature-dependent PL measurements can thus serve as an indirect means to assess the core–shell interfacial stability and the efficiency of radiative recombination. Accordingly, we analyzed the temperature-dependent PL behavior of the 1-step and 2-step ZnSe-shelled QDs.

**Figure 4:**
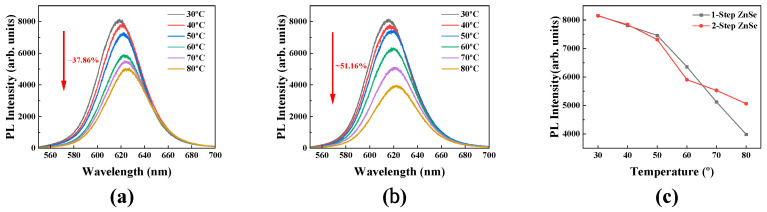
Temperature-dependent PL spectra of InP/ZnSe/ZnS quantum dots synthesized via (**a**) 2-step ZnSe shelling and (**b**) 1-step ZnSe shelling methods. The integrated PL intensity of the two samples was compared in (**c**) as a function of temperature.

The measurements were conducted in the temperature range of 30 °C to 80 °C, and a 532 nm laser was used as an excitation source. Figure 4a,b shows the PL spectra of each QD sample at different temperatures. Both samples exhibited a general decrease in PL intensity with increasing temperature, which is attributed to the trapping of charge carriers at interfacial defect states, leading to reduced radiative recombination efficiency. In addition, the red shift of the PL peak with increasing temperature can be explained by the Fan expression as a typical bandgap shrinkage phenomenon, which is universally observed in all QDs [30]. However, the 2-step ZnSe-shelled QDs maintained a higher PL intensity than the 1-step ZnSe-shelled QDs, particularly at elevated temperatures above 60 °C.

Figure 4c presents the temperature dependence of the integrated PL intensity. The 1-step ZnSe-shelled QDs showed a PL intensity reduction of approximately 51.16% at 80 °C compared to 30 °C, whereas the 2-step ZnSe-shelled QDs exhibited a smaller decrease of 37.86%, indicating a more pronounced thermal stability. Both QD samples exhibited similar PL decay trends up to 50 °C; however, a significant difference was observed in the high temperature range above 60 °C. In particular, from 60 °C to 70 °C, the 1-step ZnSe QDs showed a steep decrease of 19.39%, while the 2-step ZnSe QDs exhibited a more gradual reduction of 6.36%. Furthermore, in the 70 °C to 80 °C range, the 1-step ZnSe-shelled QDs experienced a sharp decrease of 22.26%, compared to only 8.46% reduction for the 2-step sample. Table 3 shows the reduction rate of the two types of QDs in each temperature range. The reduction rate was defined as the percentage decrease relative to the value measured at the initial temperature. Reliable operation of display devices above 60 °C is particularly important in certain applications such as automotive displays, where cabin temperatures can reach 80–90 °C during the summer. Since automotive displays are among the main target applications of QD technology, as demonstrated by the models showcased by major display companies at various exhibitions, the enhanced thermal stability of the 2-step QDs may have significant practical implications.

Zhao et al. reported that irreversible luminescence quenching in QD systems tends to initiate at elevated temperatures around 100 °C, attributing the phenomenon to thermally activated carrier migration toward surface defects or interfacial trap sites, which in turn induces nonradiative recombination [31]. In this study, similar quenching behavior was observed at a lower threshold of approximately 60 °C, likely due to the measurements being performed in thin-film form and the structural differences in the QDs. Zhao et al. also associated the PL quenching and red shift of the emission peak with thermally activated trap states. Consistent with this, the present results suggest that trap activation under thermal stress facilitates carrier loss via nonradiative pathways, thereby degrading optical performance. The escape of thermally activated carriers from the shell is also closely related to interface or shell defects, and therefore, the improved thermal stability observed in quantum dots (QDs) with a 2-step ZnSe shell indicates effective suppression of these defects.

### 3.4. Structural Characterization by Transmission Electron Microscopy and Energy-Dispersive X-Ray Spectroscopy (TEM-EDS)

**Figure 5:**
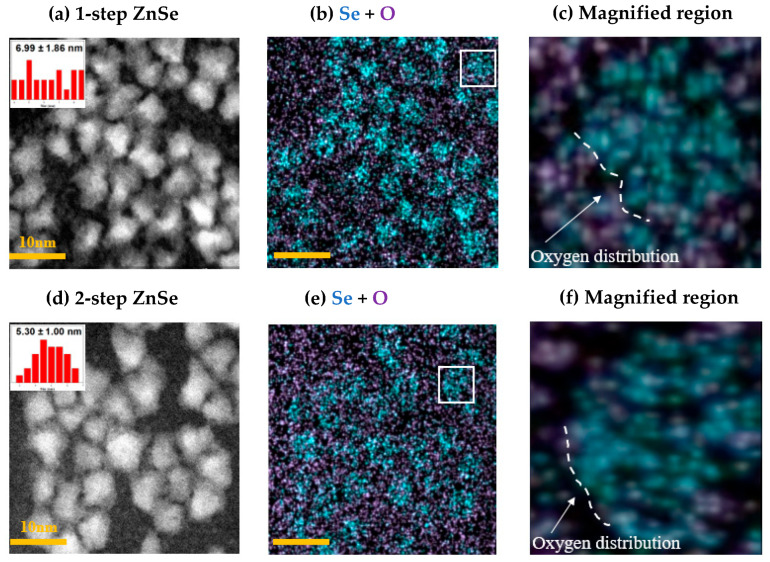
(**a**,**d**) TEM images of 1-step and 2-step ZnSe-shelled InP QDs. (**b**,**e**) EDS elemental mapping showing the overlaid distribution of selenium (cyan) and oxygen (purple), (**c**,**f**) Magnified regions from (**b**,**e**), respectively, highlighting oxygen distribution adjacent to the Se interface.

To visually validate how the differences in optical properties—observed in PLQY, TRPL, and temperature-dependent PL—are linked to structural factors at the core–shell interface, high-resolution TEM and EDS analyses were performed. Figure 5 shows the EDS elemental mapping results for the 1-step and 2-step ZnSe-shelled InP QDs. By examining the spatial distributions of key elements, including In, P, Zn, Se, and S, the shell composition and interfacial characteristics can be directly compared [32,33,34].

In the 1-step ZnSe-shelled sample, non-uniform shell growth may occur, and such structural irregularities are a major source of interfacial defects and void formation. The presence of voids can promote oxygen trapping from the environment, which in turn leads to degradation of optical properties. To assess shell uniformity, oxygen trapping due to defects, and lattice compatibility, we focused on analyzing oxygen distribution at the Se interface, which serves as a critical parameter [12,35,36].

The TEM images shown in Figure 5a,d reveal that both QD samples exhibit similar particle sizes and morphologies. Figure 5b,e presents the overlaid EDS elemental maps of selenium (Se) and oxygen, with Se used as the spatial reference for evaluating oxygen distribution. Figure 5c,f provides particle-level visualizations of oxygen localization at the Se interface. These analyses allow for a direct assessment of oxygen distribution near the Se interface, which was subsequently used to evaluate interfacial defect formation related to oxygen trapping.

In the 1-step ZnSe-shelled QDs, the oxygen signal (purple) appears closely co-localized with the Se signal (blue) near the core–shell interface. This suggests that oxygen trapping occurred in the vicinity of the Se interface, likely due to structural defects arising from non-uniform shell growth. When epitaxial matching is insufficient during shell formation, lattice defects may form at the interface between the ZnSe shell and the InP core, facilitating the incorporation of oxygen trap sites near the Se interface. These interfacial oxygen-related defects are considered detrimental to the optical performance, contributing to reduced PLQY and shortened carrier lifetime.

In contrast, the 2-step ZnSe-shelled QDs benefited from an initial thin ZnSe layer formed at low temperature, which promoted uniform shell growth in subsequent stages and mitigated interfacial defect formation. As a result, the oxygen distribution near the Se interface—clearly observed in the 1-step sample—was significantly suppressed. This trend is also supported by the quantitative data shown in Figure 5c,f. As summarized in Table 4, the oxygen-to-selenium ratio in the 1-step ZnSe QDs was approximately 104.3%, noticeably higher than the 78.3% observed in the 2-step ZnSe sample. These results provide quantitative evidence that interfacial defects and oxygen trapping are more prominent in the 1-step shelling method, while the 2-step approach effectively suppresses such structural instabilities through improved interface engineering.

### 3.5. Lattice Pattern

**Figure 6:**
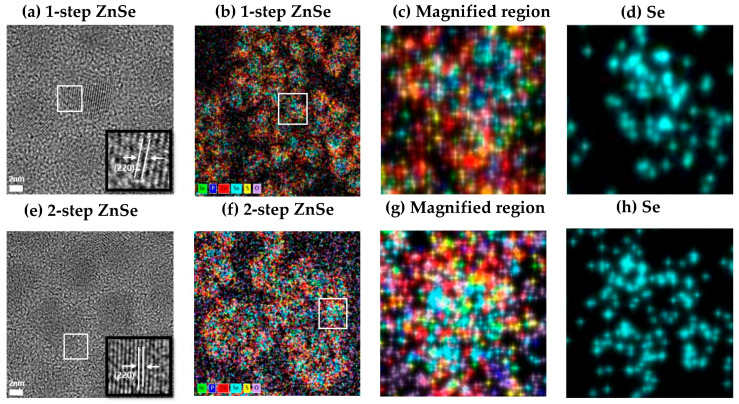
(**a**,**e**) Lattice sharpness images of InP quantum dots with 1-step and 2-step ZnSe-shelled QD samples, respectively. (**b**,**f**) EDS elemental mapping images showing the spatial distribution of In, P, Zn, Se, S, and O. (**c**,**g**) Magnified regions from (**b**,**f**), highlighting nanoscale elemental distribution at the particle level. (**d**,**h**) Selenium (Se) elemental maps extracted from the magnified regions in (**c**,**g**), respectively, visualizing the spatial distribution of Se at the core–shell interface.

Figure 6a,e shows high-resolution lattice images of QDs synthesized via the 1-step and 2-step ZnSe shelling methods, respectively. In the 1-step ZnSe-shelled QDs (Figure 6a), the lattice fringes near the core–shell interface appear relatively blurred, indicating a loss of crystallographic continuity. This observation suggests that the shell formation was non-uniform and that the structural coherence between the core and shell was not adequately maintained. Such structural instability is consistent with the previously discussed localized Se distribution and oxygen trapping and is likely a contributing factor to the increase in nonradiative recombination pathways.

In contrast, the 2-step ZnSe-shelled QDs (Figure 6e) exhibit well-defined lattice fringes extending up to the shell interface, with clear crystallographic orientation and periodicity. The magnified image further reveals a uniformly ordered lattice arrangement, indicating that stable epitaxial growth was achieved at the core–shell interface during shell formation [12,37]. This observation is also in agreement with the uniform Se distribution and the suppression of oxygen-related trap sites, ultimately supporting the observed enhancement in optical properties.

### 3.6. Selenium Distribution Uniformity

To visually investigate the origin of nonradiative recombination pathways, such as oxygen infiltration and trap formation, we analyzed the spatial distribution of Se and the corresponding lattice structure within the QDs. Figure 6b,f presents EDS elemental mapping images showing the spatial distribution of key elements—including In, P, Zn, Se, S, and O—for the 1-step and 2-step ZnSe-shelled QDs, respectively. Figure 6c,g shows magnified views at the single-particle level within the mapped region, while Figure 6d,h displays Se-only maps extracted from the same magnified regions.

In the case of the 2-step ZnSe-shelled QDs, the Se signal is broadly and uniformly distributed throughout the particle, whereas in the 1-step ZnSe-shelled QDs, Se is localized in discrete regions. This difference is attributed to variations in interfacial conditions and surface affinity during the initial stage of shell growth [38]. In the 2-step ZnSe shelling process, a thin ZnSe layer formed at low temperature serves as a nucleation template that promotes the lateral diffusion of subsequently injected Se precursors, thereby facilitating uniform shell growth across the particle. In contrast, the rapid shell growth in the 1-step process leads to localized reactions and aggregation of Se precursors, resulting in uneven distribution.

Ultimately, this difference in shell diffusivity has a direct impact on the uniformity of Se distribution, which not only affects the epitaxial compatibility of the outer ZnS shell but also correlates strongly with the overall interfacial stability and optical properties of the core–shell structure.

## 4. Conclusions

In this study, the correlation between structural stability and optical performance in InP-based quantum dots (QDs) was systematically investigated at the core–shell interface level. InP/ZnSe/ZnS double-shelled QDs were synthesized using either a 1-step or 2-step ZnSe shelling strategy on an identical InP core, and their optical properties were compared. The QDs fabricated via the 2-step ZnSe shelling method exhibited superior performance, with enhanced core–shell lattice matching, favorable TRPL decay dynamics, improved thermal photoluminescence stability, and slightly higher PLQY compared to those prepared by the 1-step method. This enhancement was attributed to the formation of an initial thin ZnSe interfacial layer at low temperature, which facilitated improved crystallinity and suppressed interfacial defects during subsequent shell growth.

As a result, the 2-step ZnSe-shelled QDs achieved a high PLQY of 95.7% and maintained 62.14% of their emission intensity at elevated temperatures up to 80 °C, demonstrating their thermal and optical robustness. Structural analyses using TEM and EDS provided direct visual evidence supporting these optical trends. In the 1-step ZnSe-shelled QDs, a high degree of oxygen localization near the Se interface was observed, with an O/Se atomic ratio of 104.3%, indicative of interfacial trap formation. In contrast, the 2-step ZnSe-shelled QDs exhibited a more uniform Se distribution and a significantly reduced O/Se ratio of 78.3%, confirming the suppression of oxygen-related trap states. Moreover, high-resolution TEM images revealed discontinuous and blurred lattice fringes at the core–shell boundary in the 1-step samples, whereas the 2-step samples displayed well-ordered and coherent lattice structures extending across the interface.

Taken together, these results demonstrate that the 2-step ZnSe shelling strategy may enhance the optical performance of InP-based QDs by improving structural coherence and mitigating interfacial defect formation. Atomic-scale structural analyses provide compelling visual and quantitative evidence for the effectiveness of this shelling approach, thereby offering valuable guidance for interface-engineering strategies aimed at developing high-performance quantum dot emitters.

## Figures and Tables

**Table 1:** Optical properties of InP/ZnSe/ZnS quantum dots synthesized by 1-step ZnSe and 2-step ZnSe shelling.

Shelling Method	Peak Wavelength (nm)	PLQY (%)	FWHM (nm)
1-step ZnSe	603.9	94.0 ± 1.1	38.0 ± 0.1
2-step ZnSe	607.6	95.7 ± 1.4	39.0 ± 0.1

**Table 2:** Double-exponential fitting parameters of TRPL decay curves for 1-step and 2-step ZnSe.

Shelling	$a_{1}$	$a_{2}$	$\tau_{1}$	$\tau_{2}$	$\tau_{avg}$
1-step ZnSe	0.28	0.72	14.34	37.19	34.20
2-step ZnSe	0.31	0.69	16.94	36.28	32.93

**Table 3:** PL intensity reduction rate in each temperature range.

Temperature Range	Reduction Rate (%) (1-Step Shelling)	Reduction Rate (%) (2-Step Shelling)
30 °C → 40 °C	4.28	3.74
40 °C → 50 °C	4.51	6.74
50 °C → 60 °C	14.73	19.24
60 °C → 70 °C	19.39	6.36
70 °C → 80 °C	22.26	8.46

**Table 4:** Quantitative analysis of Se and O elemental signals obtained from the EDS maps in Figure 5c,f. The O/Se ratio was calculated based on particle counts to evaluate the relative distribution of oxygen near Se-rich regions.

Shelling	Se Count	O Count	O/Se ratio (%)
1-step ZnSe	1425	1486	104.3%
2-step ZnSe	1247	977	78.3%

## Data Availability

The original contributions presented in the study are included in the article, further inquiries can be directed to the corresponding author.
